# Risks and benefits of additional surgery for early gastric cancer in the upper third of the stomach meeting non-curative resection criteria after endoscopic submucosal dissection

**DOI:** 10.1186/s12957-022-02780-2

**Published:** 2022-09-26

**Authors:** Sin Hye Park, Hong Man Yoon, Keun Won Ryu, Young-Woo Kim, Myeong-Cherl Kook, Bang Wool Eom

**Affiliations:** Center for Gastric Cancer, National Cancer Center, Ilsan-ro 323, Ilsandong-gu, Goyang-si, 10408 Republic of Korea

**Keywords:** Proximal gastric cancer, Non-curative endoscopic resection, Additional gastrectomy, Lymph node metastasis

## Abstract

**Background:**

Additional surgery is recommended after non-curative endoscopic submucosal dissection for early gastric cancer. However, it is not easy to recommend for tumors located in the upper third of the stomach, because it would be a total or proximal gastrectomy. This study aimed to evaluate the actual risks and benefits of additional gastrectomy for upper third tumors.

**Methods:**

We reviewed the clinicopathological data of patients who underwent total or proximal gastrectomy for early gastric cancer in the upper third of the stomach between March 2002 and January 2021. The incidence of lymph node metastasis and postoperative complications were calculated, and risk factors for lymph node metastasis were identified using logistic regression analysis. Survival rates were analyzed using the Kaplan–Meier method and log-rank test.

**Results:**

A total of 523 patients underwent total or proximal gastrectomy for early gastric cancer; 379 of them had tumors meeting the non-curative resection criteria for endoscopic submucosal dissection. The overall lymph node metastasis rate was 9.5%, and lymphovascular invasion was the only significant risk factor for lymph node metastasis (*p* < 0.001). The most common sites of lymph node metastasis were stations 1, 3, and 7, with their rates being 3.2%, 3.7%, and 3.2%, respectively. Overall and severe (Clavien–Dindo grade III or higher) postoperative complication rates were 21.1% and 14.0%, respectively, while postoperative mortality was 0.5% (2/379). The 5-year overall survival rates for patients with and without lymph node metastasis were 96.1% and 81.1%, respectively (*p* = 0.076).

**Conclusions:**

Before planning an additional gastrectomy after non-curative endoscopic resection for the upper third tumor, we should consider both the benefit of the 9.5% curability for lymph node metastasis and the risks of the 21% postoperative complications and 0.5% mortality.

## Background

Among all gastric cancers, early gastric cancer (EGC) cases are gradually increasing in Eastern Asia. According to the Korean nationwide survey on surgically treated gastric cancers, the proportion of EGC was 57.7% in 2009 and increased to 63.9% in 2019. Considering that most cases receiving endoscopic treatment have EGC, the overall proportion of EGC cases is expected to be over 75% in Korea [1]. In the 35-year surgical report of a Japanese hospital, the proportion of EGC cases increased from 28% in 1971 to 54% in 2007 [2]. This high EGC incidence was also shown to be 51.2% in the report of the Japanese nationwide registry in 2008 [3]. Given the increasing incidence of EGC, endoscopic treatments have been widely applied [4–6]. As experiences with endoscopic submucosal dissection (ESD) have been accumulated, the indication for ESD has been extended even to undifferentiated-type carcinomas less than 2 cm [7, 8]. In Korea, the total number of annual ESD cases for EGC increased to approximately 9000 in 2019 [9].

When an ESD is determined as a non-curative resection based on pathological findings, additional surgery is recommended because of a considerable risk of lymph node metastasis. The lymph node metastasis rate after non-curative ESD has been reported to be 5.7–9.3% [10–12]. If the tumor is located in the middle or lower third of the stomach, distal gastrectomy will be considered; this has a low risk of postoperative complications. However, if the tumor is located in the upper third of the stomach, total or proximal gastrectomy is mandatory, and either of these has a high risk of morbidity and long-term sequelae [13–15]. Compared with distal gastrectomy, total gastrectomy shows a higher rate of immediate postoperative complications, including anastomotic leakage, intra-abdominal abscess, and wound complications [15]. Moreover, anastomotic stricture occurs more frequently after total and proximal gastrectomies within several months [16, 17]. In terms of long-term sequelae, the incidences of nutritional deficiencies, such as severe weight loss, iron-deficiency anemia, and vitamin B12 deficiency, are significantly higher after total gastrectomy than after distal gastrectomy [18–20]. Therefore, it is not easy to recommend additional surgery for tumors located in the upper third of the stomach.

Hence, it is necessary to evaluate the actual risks and benefits of additional surgery after non-curative ESD for tumors located in the upper third of the stomach. In this study, we aimed to identify the exact incidence of lymph node metastasis and postoperative morbidity in patients who underwent total and proximal gastrectomies for EGC that met the non-curative resection criteria for ESD. The results of this study will help clinicians to inform patients and make appropriate decisions regarding additional surgery based on the actual risks and benefits involved. This information is especially important for elderly patients with comorbidities [21].

## Methods

### Patients

We retrospectively reviewed data collected on patients who underwent gastrectomy for gastric cancer between March 2002 and January 2021. A total of 523 patients underwent total or proximal gastrectomy for EGC, and 379 of them were diagnosed with EGC that met the non-curative resection criteria for ESD. Non-curative resection criteria for ESD were defined as resections that failed to meet any of the following expanded indication criteria; no lymphovascular invasion and (a) differentiated (well or moderately differentiated tubular or papillary) mucosal cancer without ulcer, (b) differentiated mucosal cancer measuring < 3 cm with ulcer, (c) differentiated cancer measuring < 3 cm with submucosal invasion (< 500µm), and (d) undifferentiated (poorly differentiated tubular adenocarcinoma, poorly cohesive carcinoma, including signet ring cell carcinoma, and mucinous adenocarcinoma) mucosal cancer measuring < 2 cm without ulcer [7, 8].

The Institutional Review Board at the National Cancer Center approved this study (no. NCC 2021–0292).

### Pathologic evaluation

A single pathologist (M.C.K.), who specialized in gastric cancer, performed the pathologic evaluation. Histological types were classified according to the World Health Organization classification and were divided into two categories according to the treatment guidelines: differentiated (papillary, well-differentiated, and moderately differentiated tubular adenocarcinoma) and undifferentiated (poorly differentiated tubular adenocarcinoma, poorly cohesive carcinoma, including signet ring cell carcinoma, and mucinous adenocarcinoma) [7, 8, 22].

The depth of invasion and lymph node metastasis were classified according to the eighth American Joint Committee on Cancer tumor-node-metastasis classification [23].

The incidence of lymph node metastasis at each station was also determined. Analysis of lymph node metastasis was performed using data from 354 patients, excluding data with low reliability.

### Surgical treatment

Total or proximal gastrectomy was performed depending on the tumor characteristics and surgeons’ decision. All surgical approaches, such as open, laparoscopy-assisted, totally laparoscopic, and robotic approaches, were included. In proximal gastrectomy, anastomosis was performed using either the esophagogastrostomy or the double tract reconstruction method.

The extent of lymph node dissection was D1 + or more according to the treatment guidelines [7, 8]. D1 + includes lymph node stations numbered 1, 2, 3a, 4sa, 4sb, 7, 8a, 9, and 11p for proximal gastrectomy and 1–7, 8a, 9, and 11p for total gastrectomy.

Postoperative complications were defined as any complication occurring within 30 days after surgery and graded according to the Clavien–Dindo classification [24].

### Follow-up

Patients were regularly followed-up every 6 months for 3 years and annually thereafter for 5 years at least after surgery. Survival status was collected from the medical records and claims data of the Korean National Health Insurance Corporation. Mortality was considered the reason for the disqualification of health insurance, while censoring was the reason for the maintenance of insurance on the date of screening.

### Statistical analysis

Categorical variables are presented as frequencies and percentages, and continuous variables are presented as means and standard deviations. Significant differences in categorical data were examined using the chi-square test or Fisher’s exact test. The Student’s *t* test or Mann–Whitney *U* test was used for continuous variables. Univariate and multivariate logistic regression analyses were performed to identify risk factors for lymph node metastasis, and the results of the logistic regression model are presented as odds ratios (ORs) with 95% confidence intervals. The survival time was calculated as the interval between the date of surgery and the last evaluation date at which the patient was alive. Survival curves were estimated using the Kaplan–Meier method and the log-rank test.

*P* < 0.05 was considered statistically significant. All statistical analyses were performed using SAS version 11 (SAS Institute Inc., Cary, NC, USA).

## Results

### Patient demographic and clinicopathological characteristics according to lymph node metastasis

The overall incidence of lymph node metastasis was 9.5% (36/379). Table 1 shows the clinicopathological characteristics of the negative and positive lymph node metastasis groups. The proportions of submucosal invasion (≥ 500µm) and the presence of lymphovascular invasion were significantly higher in the positive lymph node metastasis group. No other significant differences were observed.

**Table 1:** Clinicopathologic characteristics between negative and positive LN metastasis groups

Variables	Negative LNM group [*n* = 343 (%)]	Positive LNM group [*n* = 36 (%)]	*P* value
Age (mean ± SD) (year)	58.5 ± 11.1	58.5 ± 14.2	0.995
Sex			0.08
Male	250 (72.9)	21 (58.3)	
Female	93 (27.1)	15 (41.7)	
BMI	24.1 ± 2.8	23.7 ± 3.2	0.363
ASA			0.459
1	127 (37.1)	11 (30.6)	
2	193 (56.4)	24 (66.7)	
3	22 (6.4)	1 (2.8)	
Approach			0.132
Open	153 (44.6)	22 (61.1)	
Laparoscopic	166 (48.4)	13 (36.1)	
Robot	24 (7.0)	1 (2.8)	
Extent of gastrectomy			0.826
Total	277 (80.8)	30 (83.3)	
Proximal	66 (19.2)	6 (16.7)	
Tumor size (mean ± SD) (cm)	3.5 ± 1.6	3.7 ± 2.0	0.549
Tumor size			0.861
≤ 3 cm	154 (44.9)	17 (47.2)	
> 3 cm	189 (55.1)	19 (52.8)	
Histology			0.152
Differentiated	134 (39.1)	19 (52.8)	
Undifferentiated	209 (60.9)	17 (47.2)	
Depth of invasion			0.011
Mucosa	87 (25.4)	2 (5.6)	
SM1 (< 500 μm)	35 (10.2)	2 (5.6)	
SM2 (≥ 500 μm)	221 (64.4)	32 (88.9)	
Ulcer			0.507
Absent	275 (80.2)	31 (86.1)	
Present	68 (19.5)	5 (13.9)	
LVI			< 0.001
Absent	300 (87.5)	17 (47.2)	
Present	43 (12.5)	19 (52.8)	

*LNM* Lymph node metastasis, *BMI* Body mass index, *ASA* American Society of Anesthesiologists, *SM* Submucosa, *LVI* Lymphovascular invasion

### Risk factors for lymph node metastasis

Patient demographics such as age, sex, and BMI, and several pathological factors were included in the multivariate analysis to determine the risk factors for lymph node metastasis. The pathological factors related to lymph node metastasis are the tumor size, histology, depth of invasion, presence of ulcers, and lymph vascular invasion, which are the variables categorizing ESD criteria. In the multivariate Cox regression analysis, the lymphovascular invasion was the only independent risk factor for lymph node metastasis (OR 7.369 (CI 3.459–15.697), *p* < 0.001) (Table 2). Tumor size, differentiation, depth of invasion, and ulcer were not significant risk factors in this analysis.

**Table 2:** Multivariate analysis for LN metastasis in upper third early gastric cancer

Variables	OR	95% CI	*P* value
Age	0.996	0.963, 1.031	0.837
Sex			0.107
Male	1		
Female	1.968	0.864, 4.482	
BMI	0.959	0.843, 1.090	0.519
Tumor size			0.987
≤ 3 cm	1		
> 3 cm	0.987	0.472, 2.092	
Histology			0.2
Differentiated	1		
Undifferentiated	0.587	0.260, 1.325	
Depth of invasion			0.181
Mucosa	1		
SM1	1.565	0.2, 12.246	
SM2	3.516	0.764, 16.186	
Ulcer			0.825
Absent	1		
Present	0.889	0.312, 2.530	
LVI			< 0.001
Absent	1		
Present	7.369	3.459–15.697	

*OR* Odds ratio, *CI* Confidence interval, *BMI* Body mass index, *SM* Submucosa, *LVI* Lymphovascular invasion

### Incidence of lymph node metastasis at each station according to the tumor location

The incidence of lymph node metastasis at each station is described in Table 3. The most common sites of lymph node metastasis were stations 1, 3, and 7, with metastasis rates being 3.2% (11/347), 3.7% (12/326), and 3.2% (11/340), respectively. In the case of a tumor being located in the lesser curvature of the stomach, lymph node metastasis was detected in the lesser curvature side (stations 1 and 3) and supra-pancreatic area (stations 7, 8a, and 9), but not in the greater curvature side (stations 2, 4sa, 4sb, 4d, and 6). However, tumors located in the greater curvature and posterior wall of the stomach were associated with lymph node metastasis in both the lesser and greater curvature sides and supra-pancreatic area.

**Table 3:** Incidence of lymph node metastasis at each station according to the tumor location

LN station	Total (*n* = 354)	Tumor location			
		LC (*n* = 105)	AW (*n* = 71)	GC (*n* = 31)	PW (*n* = 147)
#1	3.2% (11/347)	3.0% (3/101)	1.4% (1/70)	6.5% (2/31)	3.4% (5/145)
#2	1.3% (4/310)	0% (0/93)	1.6% (1/64)	3.7% (1/27)	1.6% (2/126)
#3	3.7% (12/326)	3.2% (3/94)	2.9% (2/68)	3.3% (1/30)	4.5% (6/134)
#4sa	0% (0/298)	0% (0/84)	0% (0/61)	0% (0/24)	0% (0/129)
#4sb	0.3% (1/329)	0% (0/95)	0% (0/67)	0% (0/28)	0.7% (1/139)
#4d	0.7% (2/287)	0% (0/83)	0% (0/56)	3.6% (1/28)	0.8% (1/120)
#5	0% (0/269)	0% (0/82)	0% (0/47)	0% (0/28)	0% (0/112)
#6	0.4% (1/273)	0% (0/81)	0% (0/49)	0% (0/30)	0.9% (1/113)
#7	3.2% (11/340)	3.0% (3/101)	4.5% (3/67)	6.9% (2/29)	2.1% (3/143)
#8a	1.2% (4/340)	2.0% (2/99)	0% (0/69)	3.4% (1/29)	0.7% (1/143)
#9	0.6% (2/325)	1.0% (1/96)	0% (0/69)	0% (0/25)	0.7% (1/135)
#10	0% (0/92)	0% (0/33)	0% (0/15)	0% (0/12)	0% (0/32)
#11p	1.3% (4/300)	2.2% (2/91)	0% (0/62)	0% (0/24)	1.6% (2/123)
#11d	0.5% (1/184)	1.7% (1/60)	0% (0/33)	0% (0/11)	0% (0/80)
#12a	0.5% (1/189)	0% (0/57)	0% (0/38)	0% (0/18)	1.3% (1/76)

*LN* Lymph node, *LC* Lesser curvature, *AW* Anterior wall, *GC* Greater curvature, *PW* Posterior wall

### Postoperative complications

Details of the postoperative complications are demonstrated in Table 4. The overall postoperative complication rate was 21.1% (80/379) after total or proximal gastrectomy. When surgery was classified by surgical approach and extent, the postoperative complication rates in the open total, open proximal, laparoscopic/robot total, and laparoscopic/robot proximal gastrectomy were 22.9% (37/166), 44.4% (4/9), 18.4% (26/141), and 20.6% (13/63), respectively. The most common complications were ileus (5.5%), anastomotic stricture (5.0%), and leakage (4.0%). The most common systemic complication was pulmonary complication (2.4%).

**Table 4:** Postoperative complications

	Total (*n* = 379) (%)	Open TG (*n* = 166) (%)	Open PG (*n* = 9) (%)	Lapa/robot TG (*n* = 141) (%)	Lapa/robot PG (*n* = 63) (%)
Number of patients with morbidity	80 (21.1)	37 (22.9)	4 (44.4)	26 (18.4)	13 (20.6)
Local complications					
Wound	5 (1.3)	3 (1.8)	0 (0)	2 (1.4)	0 (0)
Bleeding	1 (0.3)	1 (0.6)	0 (0)	0 (0)	0 (0)
Fluid collection	9 (2.4)	7 (4.2)	0 (0)	2 (1.4)	0 (0)
Anastomotic leakage	15 (4.0)	2 (1.2)	0 (0)	7 (5.0)	6 (9.5)
Anastomotic stricture	19 (5.0)	7 (4.2)	1 (11.1)	8 (5.7)	2 (3.2)
Ileus	21 (5.5)	11 (6.6)	1(11.1)	7 (5.0)	2 (3.2)
Internal herniation	7 (1.8)	3 (1.8)	0 (0)	4 (2.8)	0 (0)
Incisional hernia	3 (0.8)	3 (1.8)	0 (0)	0 (0)	0 (0)
Systemic complications					
Pulmonary	9 (2.4)	4 (2.4)	1 (11.1)	2 (1.4)	2 (3.2)
Cardiac	1 (0.3)	0 (0)	0 (0)	1 (0.7)	0 (0)
Urinary	1 (0.3)	0 (0)	0 (0)	1 (0.7)	0 (0)
Cholangitis/cholecystitis	3 (0.8)	1 (0.6)	1 (11.1)	0 (0)	1 (1.6)
Others^a^	4 (1.1)	2 (1.2)	1 (11.1)	1 (0.7)	0 (0)
Clavien-Dindo grade					
I	11 (2.9)	6 (3.6)	0 (0)	5 (3.5)	0 (0)
II	33 (8.7)	18 (10.8)	1 (11.1)	10 (7.1)	4 (6.3)
IIIA	35 (9.2)	10 (6.0)	4 (44.4)	14 (9.9)	7 (11.1)
IIIB	14 (3.7)	9 (5.4)	0 (0)	4 (2.8)	1 (1.6)
IVA	2 (0.5)	0 (0)	0 (0)	1 (0.7)	1 (1.6)
IVB	0 (0)	0 (0)	0 (0)	0 (0)	0 (0)
V	2 (0.5)	1 (0.6)	0 (0)	1 (0.7)	0 (0)
IIIA or more	53 (14.0)	20 (12.0)	4 (44.4)	20 (14.2)	9 (14.3)
Mortality	2 (0.5)	1 (0.6)	0 (0)	1 (0.7)	0 (0)

^a^Included chylous ascites, afferent loop syndrome, splenic infarction, and constipation

*TG* Total gastrectomy, *PG* Proximal gastrectomy

The incidence of a severe complication (Clavien–Dindo grade III or higher) was 14.0% (53/379). Their proportions in the open total, open proximal, laparoscopic/robot total, and laparoscopic/robot proximal gastrectomy were 12.0%, 44.4%, 14.2%, and 14.3%, respectively. There were two cases (0.5%) of operation-related mortality after total gastrectomy (one each of open and laparoscopic).

### Long-term outcomes according to the lymph node metastasis

**Fig. 1:**
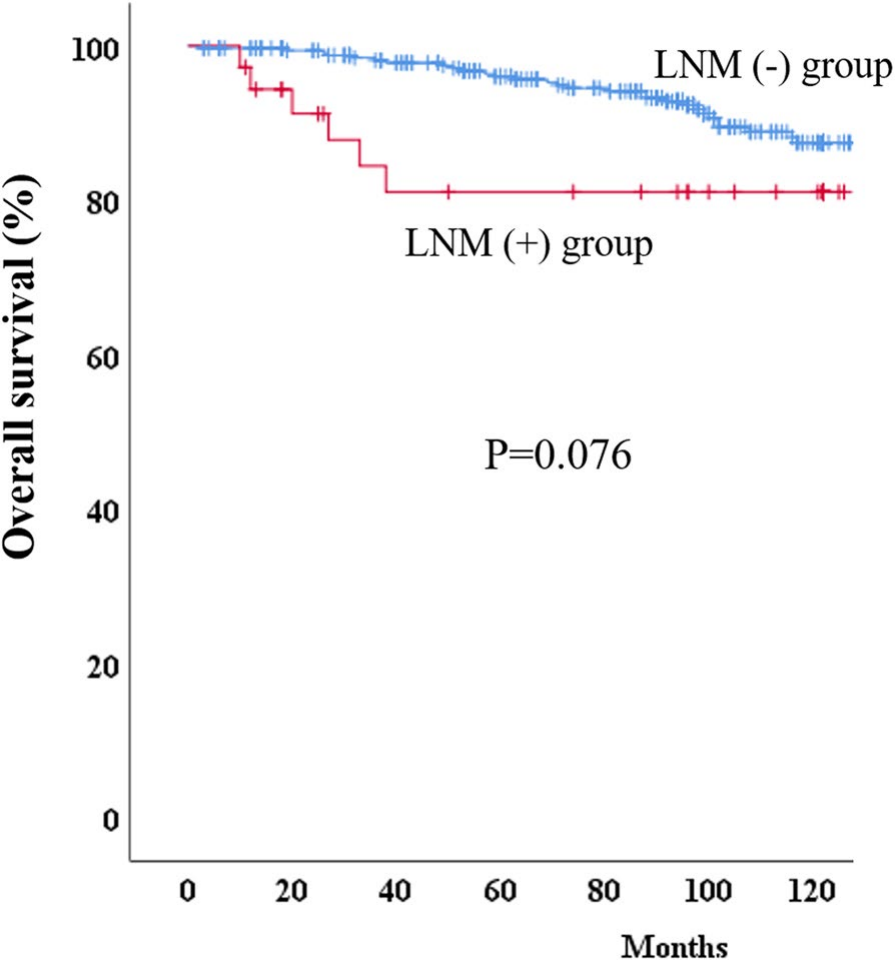
Overall survival rates in the negative and positive lymph node metastasis groups

A total of 35 (9.2%) patients died with a mean follow-up of 144 months (95% CI, 141.3–148.4). In the positive lymph node metastasis group, four (11.1%) patients had gastric cancer recurrences and died (three liver and one bone metastases). There was no gastric cancer recurrence and gastric cancer-specific death in the negative lymph node metastasis group. Deaths of other primary cancers were observed in six patients in the negative lymph node metastasis group. Overall survival was compared between the negative and positive lymph node metastasis groups, and the 5-year overall survival rates were 96.1% and 81.1%, respectively (*p* = 0.076) (Fig. 1).

## Discussion

According to a nationwide survey, the number of EGC and upper third tumor cases, among all gastric cancers, is increasing in Korea [1]. The best treatment scenario is when the tumor is located in the upper third of the stomach to meet the indications of ESD and when the resection is determined as curative. However, if the ESD is non-curative, total or proximal gastrectomy should be recommended as additional surgery. This decision is challenging due to the high risk of postoperative morbidity and long-term sequelae.

In this study, the incidence of lymph node metastasis was investigated to evaluate the oncological benefit of additional surgery. Many previous studies reported overall incidences of lymph node metastasis of tumors that met the non-curative resection criteria for ESD [10, 12, 25]. However, a few studies have focused on the upper third EGC, some of which revealed that the overall incidences of lymph node metastasis were between 7.0 and 10.1% for EGC involving the upper third of the stomach [26, 27]. Another study showed that 11.1% of cases resulted in lymph node metastasis after sentinel navigation surgery for upper third EGC < 4 cm in size [28]. One different aspect of this study from previous studies is that tumors meeting non-curative resection criteria were exclusively included. Therefore, we could identify the exact incidence of lymph node metastasis in patients who have to decide whether to have additional surgery.

For the risk assessment, we determined the postoperative morbidity and mortality after total and proximal gastrectomies for EGC. Previous studies reported a complication rate of 8.0–11.6% after laparoscopic distal gastrectomy; however, it was doubled (15.1 ~ 26.9%) after laparoscopic total gastrectomy [14, 16, 29]. This study also showed similar overall and major complication rates of 21.1% and 14.0% after total and proximal gastrectomies, respectively. Moreover, severe body weight loss can induce decreased stamina and physical activity, which results in poor quality of life [18, 30].

Complication patterns and rates can differ according to the anastomosis method after proximal gastrectomy. Esophagogastrostomy has significantly higher incidences of reflux esophagitis and anastomotic stenosis compared to double tract reconstruction. Some patients with esophagogastrostomy can be on proton pump inhibitors for a long time [31, 32]. In contrast, double tract reconstruction has similar incidences of reflux esophagitis or anastomotic stenosis when compared with total gastrectomy. However, surveillance of the remnant stomach can be difficult because of a long or twisted Roux-limb, which is a critical drawback of the double tract reconstruction method, particularly in countries, such as Korea, with a high prevalence of gastric cancer. Therefore, many surgeons are still hesitant to perform proximal gastrectomy.

The anatomical location of lymph node stations was defined according to the Japanese classification of gastric carcinoma [33]. In this study, lymph node metastasis was mainly observed in the lymph nodes located on the lesser curvature side (3 and 7), which is in accordance with the findings of previous studies [26, 27]. Furthermore, we determined the incidence of lymph node metastasis at each station according to the tumor location, considering the possibility of limited lymph node dissection and local resection to avoid total or proximal gastrectomy. However, although the incidence was low, supra-pancreatic lymph node metastasis was still observed in all tumor locations and limited lymph node dissection should be very cautious.

Comparing survival outcomes between patients who underwent total or proximal gastrectomy and those who did not would be an ideal study design. However, this study included only patients who underwent additional surgery; therefore, we could not identify the actual survival benefit of the additional surgery. Instead, this study showed a considerable difference in survival between the negative and positive lymph node metastasis groups. Although the difference was insignificant (*p* = 0.076), a 15% survival difference cannot be ignored in patients with EGC. Therefore, additional surgery should be considered for patients with a high risk of lymph node metastasis, especially those with lymphovascular invasion.

This study has several limitations. First, this study included patients treated during the learning curve period for the laparoscopic procedure. Therefore, the postoperative complication rate was comparatively higher than that in clinical trials in which standardized surgery was performed. Second, the results of long-term sequelae, such as weight loss and nutritional deficiency, of total and proximal gastrectomies were not analyzed because of the lack of data. Third, this was a retrospective study and some lymph node metastasis data were insufficient for analysis. Fourth, the data came from a single high-volume center, and hence, there is a possibility of selection bias.

## Conclusion

Before planning an additional gastrectomy after non-curative ESD for upper third tumor, we should consider both the benefit of the 9.5% curability for lymph node metastasis, and the risks of the 21% postoperative complications and 0.5% mortality.

## Data Availability

The datasets used and/or analyzed during the current study are available from the corresponding author.

## Abbreviations

EGD: Early gastric cancer
ESD: Endoscopic submucosal dissection
OR: Odds ratio
